# What is your Diagnosis?

**DOI:** 10.5812/kmp.iranjradiol.17351065.3152

**Published:** 2011-11-25

**Authors:** Leila Aghaghazvini, Hashem Sharifian, Habib Mazaher, Shirin Aghaghazvini

**Affiliations:** 1 Department of Radiology, Shariati Hospital, Tehran University of Medical Sciences, Tehran, Iran; 2 Department of Radiology, Amiralam Hospital, Tehran University of Medical Sciences, Tehran, Iran; 3 Advanced Diagnostic and Interventional Radiology Research Center (ADIR), Imam Khomeini Hospital, Tehran University of Medical Sciences, Tehran, Iran

A 10 year-old girl who was referred with snoring and dyspnea. On physical examination severe hypertrophy of adenoid tissue is noted. Note the following computed tomography (CT) scan and magnetic resonance imaging (MRI).

**Figure 1:**
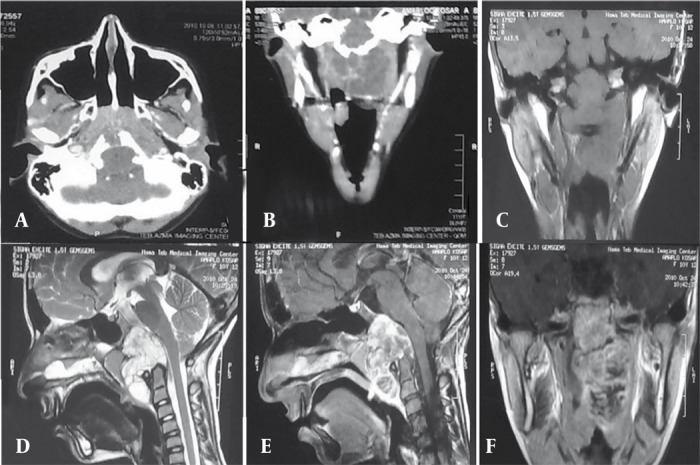
A and B, Contrast CT Scan Reveals a Heterogeneous Enhancing Mass in the Nasopharyngeal Region With Destruction of the Posterior Portion of Clivus and Bulging to Foramen Magnum . C-F, MRI With and Without Contrast Shows a Large Heterogeneous Signal Enhancing Mass in the Retropharyngeal Space of the Nasopharynx Extending Anteriorly to the Uvula and Posteriorly to Foramen Magnum Compressing the Medulla and Cord With Involvement of the Posterior Portion of the Clivus.

## Diagnosis: Clival Chordoma

Chordomas constitute approximately 1% of intracranial tumors and 4% of all primary bone tumors. Most of them occur in the sacrum (50%), and the clivus and basiosphenoid (35%) in the midline [1]. These tumors originate from the notochordal remnant and develop along the craniovertebral axis. They are classified histologically as a benign tumor (slow growing), but clinically as a malignant tumor (invasive behavior relative to adjacent structures) [2][3].

Most of the clival chordomas frequently present during the fifth decade in adults and are very rare in childhood [1][2][3][4][5]. (less than 5% in patients younger than 20 years). In the young and children, chordomas are classified into two groups:

1) Younger than 5 years

2) Between 5 and 20 years of age

They behave differently in prognosis, histopathology, metastasis rate and therapeutic management [3]. The aggressiveness and metastasis is more often seen in patients younger than 5 years of age [3][4]. Surgical removal is a very effective treatment for intracranial chordomas; however, complete treatment of skull base chordomas is very difficult and complex [3][4][5]. This case was a 10 year-old girl who was referred with snoring and dyspnea from 6 months ago. On physical examination, severe hypertrophy of the adenoid tissue is noted. Otherwise, the clinical exam was unremarkable. On CT scan, there is an asymmetric large heterogeneous enhancing soft tissue mass in the nasopharynx with mild destruction of the posterior aspect of the clivus bulging to magnum foramina in the midline. On MRI, there is a heterogeneous signal soft tissue mass in the retropharyngeal space of the nasopharynx mainly on the left side enhancing heterogeneously, extending antero-inferiorly to the oropharynx and uvula and posteriorly to magnum foramina with involvement of the clivus and atlas body and pressure on the medulla and cord. Regarding the patient’s age, history, CT and MRI findings our diagnosis was undifferentiated adenocarcinoma; however, lymphoma and chordoma were the differential diagnoses. Biopsy revealed chordoma. The patient underwent surgery and after 6 months no recurrence was detected. Our case was interesting because of the aggressive presentation of chordoma and rarity in childhood.
